# Donor Vδ1^+^ γδ T cells expand after allogeneic hematopoietic stem cell transplantation and show reactivity against CMV-infected cells but not against progressing B-CLL

**DOI:** 10.1186/2162-3619-2-14

**Published:** 2013-05-11

**Authors:** Immo Prinz, Kristina Thamm, Matthias Port, Eva M Weissinger, Michael Stadler, Ildar Gabaev, Roland Jacobs, Arnold Ganser, Christian Koenecke

**Affiliations:** 1 Institute of Immunology, Hannover Medical School, Hannover, Germany; 2 Department of Hematology, Hemostasis, Oncology and Stem-Cell Transplantation, Hannover Medical School, Hannover, Germany; 3 Institute of Virology, Hannover Medical School, Hannover, Germany; 4 Department of Clinical Immunology and Rheumatology, Hannover Medical School, Hannover, Germany

**Keywords:** B-CLL, Vδ1^+^ T lymphocytes, CMV reactivation, Allogeneic stem cell transplantation

## Abstract

γδ T lymphocytes play an important role in immune reactions towards infections and malignancies. In particular, Vγ9^–^Vδ1^+^ T lymphocytes are thought to play protective antiviral roles in human CMV infection. Recently, Vδ1^+^ T lymphocytes were proposed to also have anti- B-CLL reactivity. Here we report a case of 48-year-old man who received allogeneic stem cell transplantation for progressive B-CLL. Within one year after transplantation, lymphoma relapsed despite a dramatic increase of Vδ1^+^ T cells in the patient’s blood. *In vitro* killing assays revealed activity of patient’s γδ cells against CMV target cells, but not against the relapsing lymphoma-cells. This argues for a contribution of Vδ1^+^ cells in the immune reaction against CMV reactivation, but does not support a strong correlation of expanded Vδ1^+^ T cells and favorable disease outcome in B-CLL patients.

## Background

Several types of T lymphocytes bearing γδ T cell receptors (TCR) are currently investigated as potential anti-cancer agents in cell-based immunotherapy. Recently, Vδ1^+^ T lymphocytes were shown to be cytotoxic to B-CLL-derived cell lines [1] and it was proposed that Vδ1^+^ T lymphocytes may contribute to limiting disease progression in B-CLL patients [2]. Activated Vδ1^+^ T lymphocytes can produce inflammatory cytokines such as TNF-α and IFN-γ and can be cytotoxic. Both cytokine release and cytotoxicity of Vδ1^+^ T lymphocytes are at least in part mediated through NKG2D receptor, which recognizes the stress-induced ligands MIC-A, MIC-B, and ULBP3 on target cells. At the same time, Vδ1^+^ T lymphocytes are implicated in the human immune response to cytomegalovirus infection [3-7]. Patients receiving allogeneic stem cell transplantation as treatment for CLL often encounter life-threatening viral infections in the post-transplant period due to strong immunosuppression. Thereby, Vδ1^+^ T lymphocytes expand during post-transplant CMV reactivation and likely participate in the immune response to CMV [3]. It has been suggested that during immune response to CMV both γδ TCR cross-reactivity and NKG2D ligands confer cross-protection against tumor cells [8-10]. Here we report a case of fulminant CMV reactivation after second allogeneic stem cell transplantation as treatment for CLL. Despite expansion of donor Vδ1^+^ T lymphocytes up to 25% of all circulating peripheral blood lymphocytes, no reactivity against relapsing B-CLL could be observed.

## Case presentation

**Figure 1:**
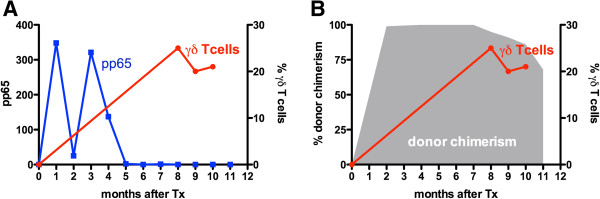
**Time course of γδ T cell expansion after transplantation (Tx).** γδ T cell frequencies among total lymphocytes are displayed in relation to virologic responses (**A**) and in relation to donor chimerism (**B**). Virologic responses were measured by pp65 antigen detection in the patient’s blood (pp65 positive per 400.000 leukocytes analysed). For determination of donor chimerism, microsatellite variable-number-of-tandem repeat of peripheral blood leukocytes was analyzed using standard techniques [11].

A 48-year-old patient was diagnosed with stage I (Rai classification) CLL in 2002 with unmutated heavy chain immunoglobulin. After 4 months of observation, he required conservative immunochemotherapy for progressive leukocytosis and lymphadenopathy (six cycles Fludarabine). In 2006, he underwent allogeneic hematopoietic stem cell transplantation from his HLA-identical brother. This led to normalization of blood counts, regression of adenopathy, and a 6-months progression-free interval. Donor and recipient were CMV^+^, however, reactivation was not observed. In March 2007, due to declining donor chimerism, he was given donor lymphocyte infusions which, however, could not prevent clinical relapse of CLL four months later. Several cycles of immunochemotherapy (Rituximab alone or in combination with Fludarabine or Bendamustine) and donor lymphocyte infusions led to stabilization for about one year, when a second relapse of B-CLL occurred. In October 2011, after successful re-induction, the patient again underwent additional allogeneic hematopoietic stem cell transplantation, now from an unrelated donor. Main complications after this second allogeneic transplantation were repetitive CMV reactivations, which were treated with four cycles of antiviral therapies including 2x Ganciclovir, Foscarnet, and Cidofovir for CMV treatment. CMV reactivation resolved in March 2012. No CMV specific CD8^+^ donor T cells could be observed by tetramer staining (data not shown), but frequencies of donor γδ T lymphocytes rose up to 25% of all circulating peripheral lymphocytes (Figure 1A). It is likely that T lymphocytes expanded during post-transplant CMV reactivation and participated in the immune response to CMV [3]. Of note, the patient showed no CMV reactivation after the first allogeneic transplantation, and frequencies of γδ T lymphocytes had not increased (3% and 4% of peripheral lymphocytes measured at 11 months and 1 month before second transplantation, respectively). Despite expanded donor γδ T cell counts, we observed a concomitant decline of donor chimerism and progressive lymphadenopathy (Figure 1B).

**Figure 2:**
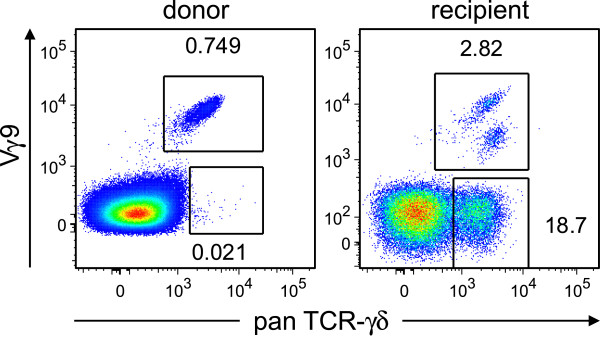
**Vδ9**^**– **^**γδ T cells expanded in the patient’s blood after transplantation.** Flow cytometric comparison of donor lymphocytes (left panel) with donor γδ T cells recovered from the patient (right panel) 9 months after transplantation. Samples were stained with PE-conjugated anti- pan TCR γδ (clone 11 F2) and FITC-conjugated anti-Vδ9 (Beckman Coulter cat. # PNIM1463). Numbers next to quadrants indicate percentage of gated Vδ9^+^ (upper gate) and V9δ^–^ (lower gate) γδ T cells among all lymphocytes.

**Figure 3:**
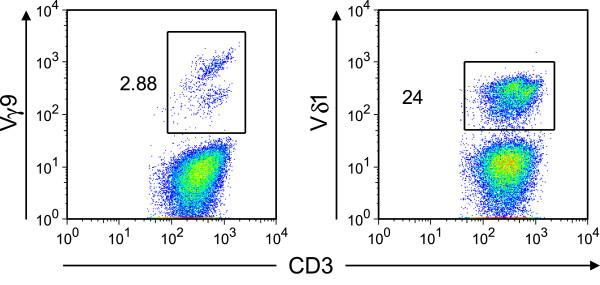
**Expanded γδ T cells in the patient’s blood are Vδ1**^**+ **^**γδ T cells.** Further flow cytometric characterization of donor γδ T cells recovered from the patient 9 months after transplantation. Ficoll-purified blood samples were stained with PE-Cy7-conjugated anti CD3 (BD bioscience, clone SK7), PC5-conjugated Vδ9 (Beckman Coulter cat. # PNA63663), and FITC-conjugated Vδ1 (Thermo Scientific, clone TS8.2, cat. # EN-TCR2730). Numbers next to quadrants indicate percentage of gated Vδ9^+^ (left panel) or Vδ1^+^ (right panel) γδ T cells among all CD3^+^ lymphocytes.

**Figure 4:**
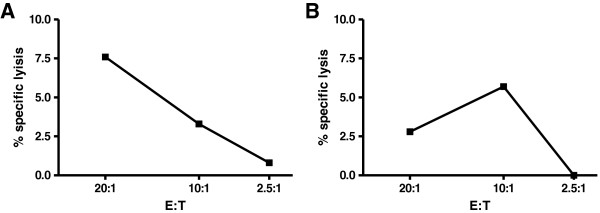
**Cytotoxicity assay of patient’s γδ T cells.** Specific lysis of sorted γδ T cells versus (**A**) CMV-infected human foreskin cells or (**B**) versus patient’s B-CLL. Total γδ T cells were sorted from patient’s blood samples using PE-conjugated anti pan γδ TCR (clone 11 F2) after Ficoll separation using a FACS Aria cell sorter 9 months after transplantation. At the same time, B-CLL cells were sorted according to size and for positive signals using PerCPCy5.5-conjugated CD19 (clone 5J25C1) and PE-Cy7-conjugated CD20 (clone L27). Cytotoxicity assay: ^51^chromium (^51^Cr) release cytotoxicity assay was performed by using sorted γδ T cells as effectors and either (**A**) CMV-infected primary human foreskin fibroblasts or (**B**) autologous tumor cells as targets. After ^51^Cr-labeling, target cells were washed and resuspended in medium (RPMI1640 supplemented with 10% foetal calf serum, 100 U/ml penicillin/streptomycin, 1 mM sodium pyruvate, and 2 mM glutamine). 100 μl of effector cells (1×10^6^/ml – 1.25×10^5^/ml) were pipetted in triplicates at three effector to target (E/T) ratios (20:1, 10:1, and 2.5:1) in V-bottom microtiter plates containing 50 μl of target cell solution (1×10^5^/ml). The assay was incubated for 20 hours. The plates were centrifuged and 25 μl of cell-free supernatants were harvested. Specific cytotoxicity was measured by determining released ^51^Cr. Background values were determined by incubating target cells without effector cells. Maximal values were obtained by lysing target cells with Triton X-100. Specific lysis was calculated by: *experimental release - spontaneous release / maximum release - spontaneous release x 100.* Generation of positive control target cells by HFF infection: HFF were cultured in Dulbecco’s modified Eagle’s medium supplemented with 10% fetal calf serum (FCS) and antibiotics. Cells were infected with HCMV TB40/E UL11-V5 mutant [12] at an m.o.i. of 1 for 120 h prior to *in vitro* killing assay.

Next, we sought to investigate the source, phenotype and reactivity of the expanded γδ T lymphocytes in more detail. First, we compared γδ T lymphocytes among donor and recipient lymphocytes. While almost all γδ T cells in peripheral blood of the stem cell donor were Vδ9^+^, the vast majority (88%) of donor γδ T cells recovered from the patient at 8.5 months after second transplantation were Vδ9^–^ (Figure 2). We suspected that the expanded T lymphocytes were Vδ1^+^ cells, which are known for CMV-reactivity [6]. Specific flow cytometric analysis confirmed that indeed 24% of all CD3^+^ lymphocytes expressed the Vδ1^+^ chain on their cell surface (Figure 3). Since donor chimerism had declined in spite of expanded donor γδ T cell counts, we tested the reactivity of FACS-sorted γδ T lymphocytes from the patient against relapsing B-CLL. At the same time, *in vitro* killing of CMV-infected human foreskin fibroblasts (HFF) was assessed (Figure 4). Interestingly, freshly *ex vivo* isolated γδ T lymphocytes from the patient, of which > 80% were Vδ1^+^Vδ9^–^ cells, showed a titratable specific lysis of CMV infected human fibroblasts, but not against his own freshly sorted leukemia cells. It is tempting to speculate that the patient’s B-CLL was not recognized because it expressed too low levels of NKG2D ligands such as MIC-A, MIC-B, or ULBP-proteins on its cell surface. Indeed, flow cytometric revealed lacking expression of these proteins on the cell surface of the patient’s B-CLL cells (data not shown).

## Conclusions

Our observations support the view that reactivity against CMV-infected cells was the driving force for Vδ1^+^ γδ T cell expansion in this patient. Previously, such expansion was neither observed in the stem cell donor’s peripheral blood nor in the patient’s blood before second transplantation. This argues for a contribution of Vδ1^+^ cells against CMV reactivation after transplantation, which is in line with recent literature [3]. However, the expanded Vδ1^+^ cells were unable to kill autologous tumor cells *in vitro* and likely also *in vivo* as the lymphoma was progressing. Taken together, our findings do not match the suggested strong correlation of expanded Vδ1^+^ T cells and favorable disease outcome in untreated B-CLL patients [2].

## Consent

Written informed consent was obtained from the patient for publication of this case report.

## Competing interests

The authors declare that they have no competing interests.

## Authors’ contributions

CK, MS and AG were responsible for the clinical management of the patient. CK, KT, IP, MP performed FACS-staining/sorting and -analysis. RJ and IG performed cytotoxicity assays. EM analyzed CMV specific CD8^+^ T cells. CK and IP were responsible for data acquisition and wrote the manuscript. All authors read and approved the final manuscript.
